# What Is the Hypoplastic Left Heart Syndrome?

**DOI:** 10.1177/21501351241232074

**Published:** 2024-03-13

**Authors:** Robert H. Anderson, Diane E. Spicer, Adrian Crucean

**Affiliations:** 1 Biosciences Division, Newcastle University, Newcastle-upon-Tyne, UK; 2 Heart Institute, Johns Hopkins All Children's Hospital, St. Petersburg, FL, USA; 3 Department of Paediatric Cardiac Surgery, Birmingham Women's and Children's Hospital, Birmingham, UK

**Keywords:** mitral valve, aortic root, cardiac pathology, functionally univentricular heart, hypoplastic left heart complex

In a recent issue of the journal, we suggested that the criteria for inclusion in the so-called “hypoplastic left heart syndrome” should include an intact ventricular septum. Initially submitted as a Letter to the Editor, the process of review of our manuscript generated significant dismay among our referees. Hence, the Editor suggested a better alternative would be to publish our viewpoint as a brief communication, with a counterpoint from those dissenting. Both communications are now published in the final issue of the journal for 2023. The alternative view is strong on opinion but short in terms of evidence. We read that the authors “strongly believe that the subtype of HLHS with ventricular septal defect in the setting of mitral atresia and aortic stenosis (or mitral stenosis and aortic stenosis) fits within the extant IPCCC and ICD-11 definition of HLHS.”^1^ This is hardly surprising when extant definitions offer no details regarding the integrity of the ventricular septum. We had anticipated that, in their counterpoint, the authors would provide the evidence as to why cases with deficient ventricular septation should be included.

As we read their response, it seems their reason might be because we had described the syndrome as representing a “spectrum of disease.” This is true. But our spectrum was restricted to lesions attributable to *acquired* disease occurring in fetal life. We had suggested exclusion of cases with deficient ventricular septation simply because such cases would imply causation during *embryonic*, rather than fetal, life. In their response, our colleagues have commented on etiologic theories, suggesting that none have been fully established. We would encourage them to review the recent studies performed at the Hospital for Sick Children in Toronto. By restricting flow through the mitral valve, the investigators have simulated the pathological findings of the syndrome both in mice and lambs.^2,3^ Like ourselves, the Canadian investigators stress the significance, as part of their models, of the integrity of the ventricular septum.

The evidence underpinning our own viewpoint came from our investigation of cases previously diagnosed by pathologists and held in archives of autopsied hearts. Maurice Lev, one of the giants of cardiac pathology, when establishing his own archive, had initially included examples in the syndrome with deficient ventricular septation. With increasing experience, he retained only those with intact ventricular septums. We have examined many of the hearts collected and diagnosed by Lev, along with the hearts gathered by Lodewyk Van Mierop, and now kept in the archive at the University of Florida in Gainesville. We have also studied the hearts now retained in the archive of Birmingham Children's Hospital in the United Kingdom. In none of these archives did we find examples with deficient ventricular septation included in the category of “hypoplastic left heart syndrome.” Our colleagues, in contrast, base their arguments on their clinical experience, the availability of previous categorizations, and on the information available from databases. But the entries in the databases are based on the previous categorizations, which to no small extent have been formulated by our colleagues themselves. Our question remains, therefore, as to whether the definitions for a given cardiac phenotype should be based on the best anatomical information available, or on the basis of previous experiences?

Disputation, nonetheless, is the life blood of science. As new information comes to hand, and as we digest and interpret the new information, it is entirely appropriate that our definitions change in accordance with our current conclusions. We await with interest, therefore, the evidence on which our colleagues have based their own interpretation of their suggested phenotypes. In this regard, in the review offered by Tchervenkov and colleagues in the initial supplement on the syndrome,^4^ a Table is provided suggesting that, of 24 suggested variants of the syndrome, 14 include deficient ventricular septation. For several combinations, cases are proposed to exist with either restrictive or nonrestrictive ventricular septal defects. Examples with deficient ventricular septation do exist, but are rare. We would not include them in our categorisation of hypoplastic left heart syndrome, the more so since, when found with a non-restrictive defect, it is arguable that the left ventricle is no longer “hypoplastic”.

**Figure 1.**
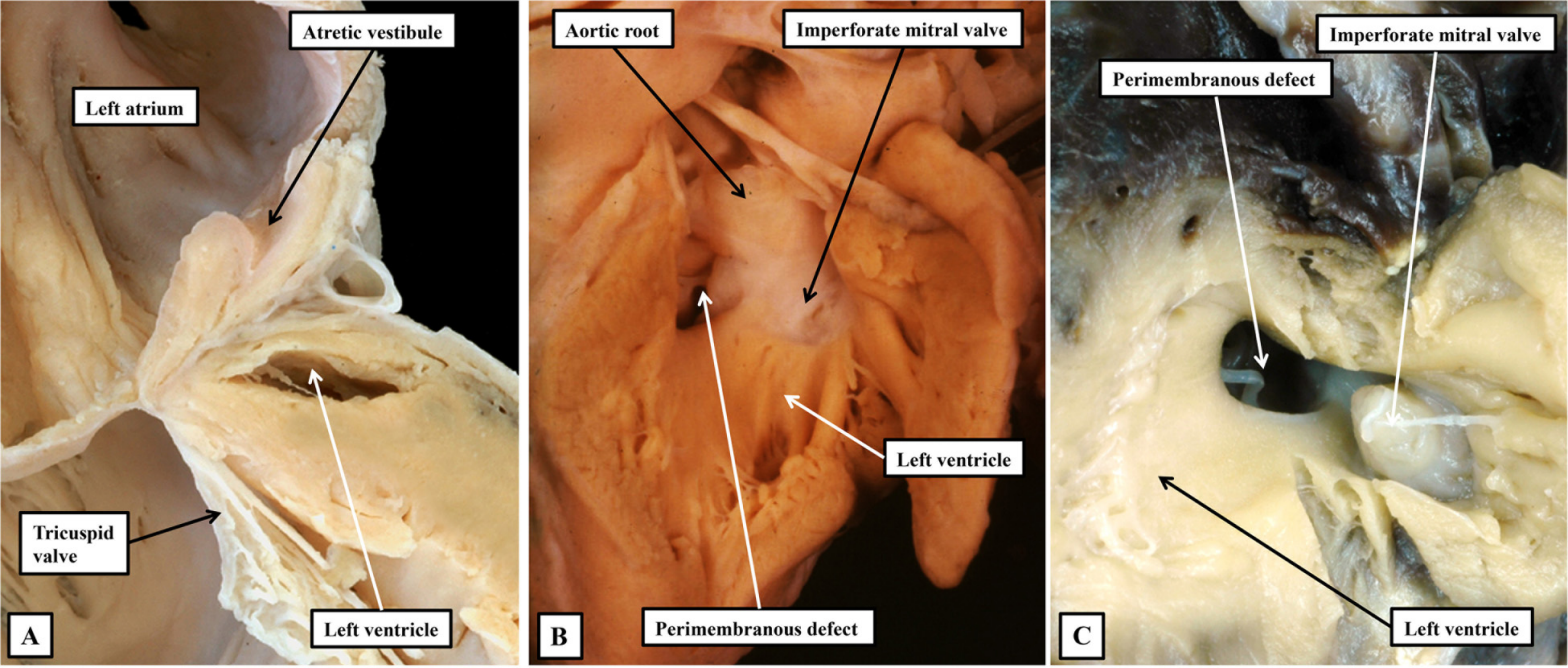
The images show the phenotypic features of mitral atresia. (A) A close-up of the left atrioventricular junction in a heart with hypoplastic left heart syndrome in the setting of combined aortic and mitral atresia. Although the left ventricle is diminutive, there is a track from the atretic vestibule of the left atrium toward the hypoplastic ventricular cavity. We interpret this feature to show the remnants of the initial left atrioventricular connection. (B and C) Examples of mitral atresia with patent aortic root. (B) The aorta arises from the hypoplastic left ventricle, whereas in the heart shown in (C), there was double outlet from the right ventricle. Both hearts show imperforate mitral valves. Using current definitions, the heart in (B) would be included in the category of “HLHS with VSD,” whereas the heart shown in (C) would not. This seems illogical, the more so when the heart shown in (B) would be grouped along with the heart shown in (A) as a variant of hypoplastic left heart syndrome. If we are to discover the genetic background of these lesions, we submit it will be necessary to take note of all details, but in particular the integrity of the ventricular septum. HLHS, hypoplastic left heart syndrome; VSD, ventricular septal defect.

Our colleagues then suggest that division of the syndrome on the basis of ventricular septation is already “described and reported in the majority of the medical literature.”^1^ It is surprising, therefore, to find that the manuscript authored by several of our colleagues, describing their experience in Gainesville, provided no details on the presence or absence of ventricular septal defects.^5^ Nor was the significance of the ventricular septum included in the review providing an overview of classification and morphology of the syndrome.^6^ The suggested groupings, however, did include the phenotype of aortic stenosis with mitral atresia. To the best of our knowledge, this combination exists only in the setting of deficient ventricular septation. It has been well described as one of the variants of mitral atresia with patent aortic root, this representing another spectrum that includes double outlet right ventricle with mitral atresia (Figure 1A and B).^7^ The latter lesion, however, is excluded by our colleagues from inclusion in the syndrome.^1^ Since they place such emphasis on spectrums, this does not seem logical.

The emphasis placed on mitral atresia, nonetheless, is pertinent when questions are raised as to whether the syndrome is, indeed, an acquired disease of fetal life. Specifically “this argument cannot be true for all variants of HLHS, because, in some cases, mitral atresia occurs early in development, with a nearly absent left ventricular chamber.”^1^ The type of atresia that occurs early in life is due to absence of the atrioventricular connection. When found in the setting of the intact ventricular septum, the atresia was shown to be due to obliteration of the initially patent left atrioventricular connection.^8^ Our own observations now support this viewpoint (Figure 1C). And as we have emphasized, the evidence now exists to show that reduction of flow through the mitral valve can induce the spectrum in the setting of an intact ventricular septum.^2,3^ A reason for including mitral atresia with patent aortic root, however, according to our colleagues, is because the lesion “is treated with the same interventions as other subtypes of HLHS.”^1^ We would direct them to the statement made by Tchervenkov and colleagues that “one cannot define anatomy, morphology, and nomenclature of a cardiac phenotype by the type of treatment used, as treatment may evolve over time.”^4^ There is, of course, nothing wrong with changing one's mind. When confronted with the evidence substantiating the need for change, we have changed our own opinions on several occasions. We are ready to change yet again when presented with evidence, as opposed to opinion, that hearts with deficient ventricular septation should be included within the hypoplastic left heart syndrome.
